# Fostering *in situ* conservation of wild relatives of forage crops

**DOI:** 10.3389/fpls.2023.1287430

**Published:** 2023-10-30

**Authors:** Christina Kägi, Blaise Petitpierre, Philipp Meyer, Yvonne Lötscher, Stefan Eggenberg, Sylvain Aubry

**Affiliations:** ^1^ Federal Office for Agriculture (FOAG), Federal Department of Economic Affairs, Education and Research EAER, Bern, Switzerland; ^2^ Infoflora, c/o Botanischer Garten, Bern, Switzerland

**Keywords:** crop wild relatives, grassland, forage crops, genetic diversity, *in situ* conservation, species distribution modelling (SDM)

## Abstract

Most plant conservation strategies generally overlook the intra-specific genetic diversity of crop gene pools. Focusing on forage crops and their wild relatives, we present a novel approach to address the conservation of these species on meadows. Two-thirds of Swiss agricultural land is green land, mostly used for forage purposes, and their genetic diversity is being threatened. We focused here on eight plant associations gathering at least 18 taxa considered priority crop wild relatives of forage crops. Since 2020, about 1,217 high-quality surfaces (representing 1,566 hectares) nationwide have been integrated into an innovative auction-based policy instrument dedicated to conserving these populations. Here, we report the benefits and hurdles of implementing this bottom-up approach and try to estimate the quality of conservation of the forage plants’ CWR gene pool. Although we focus on the Swiss case, our approach to *in situ* conservation offers opportunities to effectively guide conservation in other contexts. We also discuss possible ways to improve CWR conservation policy, particularly the need to better consider the populations and habitat levels.

## Introduction

According to the latest International Union for Conservation of Nature (IUCN) red list, about 35% of species are threatened with extinction (IUCN, 2023). While networks of protected areas are in place and recognized by the recently agreed Kunming-Montreal Global Biodiversity Framework, these measures appear not sufficient to maintain a minimum level of genetic diversity (Newbold et al., 2015; Tobón-Niedfeldt et al., 2022; Exposito-Alonso, 2023), particularly given global climate change (Li et al., 2006; Schwager and Berg, 2019; Ranius et al., 2023). Thus, innovative approaches are needed to extend the protection measures in species and valuable areas to preserve, but not necessarily considered a very high priority in conservation planning. From this perspective, progenitors of cultivated species, referred to as Crop Wild Relatives (CWR) are particularly representative. CWR taxa that are related to crops used for food, feed, and more generally agriculture, do not necessarily represent the taxa that are the most threatened, but preserving their intra and interspecific diversity is essential for the future of breeding (Dempewolf et al., 2017). In addition, the conservation of crops themselves often focused on the discrete taxa or units (like in the IUCN red list) and less on population diversity and evolution (Coates et al., 2018). In recent years, the conservation planning of CWR has substantially improved (Khoury et al., 2013; Castañeda-Álvarez et al., 2016). This planning usually gathers both *ex situ* measures (genebanks) and the identification of priority sites for *in situ* conservation (Teso et al., 2021). Beyond nature reserves, it has been shown that an important part of the CWR distribution is distributed in the agricultural area (Petitpierre et al., 2023). Efforts to identify gaps in the CWR collections concentrate on specific genetic diversity, focusing primarily on “gene pools” and “taxon groups” (Maxted et al., 2006). However, addressing directly the intra-specific diversity remains essential, particularly to breeders.

CWRs of forage crops often relate to common (not threatened) genera (Agroscope, 2023). A peculiarity of these species is their economic value being tightly associated with the various plant associations in which they are growing, and not, as most other CWR are, exclusively to one taxon (Rubio Teso et al., 2021). Therefore, any efficient measure to conserve both intra and inter-specific diversity of CWR of forage taxa must take into consideration the association’s level. This is a serious challenge both in terms of spatial and temporal monitoring, as well as for designing appropriate conservation measures. Here we present an innovative program of measures coupled with subsidies that have been deployed to take into account the challenges of CWR of forage crops spatially and timely.

Agricultural policy frames a large portion of agriculture-related environmental pressures and is intertwined with environmental policy (Mattison and Norris, 2005). In Switzerland, agriculture covers a third of the country (Guntern et al., 2012). Meanwhile, agri-environmental subsidies, which represent about 15% of the total subsidies allocated for agriculture nationally (Walter et al., 2013), have a central role to play in modulating these pressures. The *in situ* conservation instrument of CWR of forage crops presented here (hereafter the “*in situ* program”) is a very minor part of the complex regime of measures implemented over the last thirty years to promote multifunctional agriculture. Most of the subsidies targeting environmental objectives so far have been following cross-compliance schemes: the farmers are paid based on the proof of ecological performances they provide. More precisely, 7% of the land is considered an “ecological compensation area” (ECAs, Jarrett and Moser, 2013). It is estimated that 98% of farms are now complying with these standards. Since 2014, ECAs have been divided into two categories (a basic “quality 1” and an enhanced “quality 2” level) depending on species richness and localization. ECA-associated measures distinguish two distinct levels of qualities that entail species richness, limited fertilization, pesticide use, crop rotation and animal welfare. On top of ECAs that are within agricultural surfaces (fields, groves, grasslands), summer grazing surfaces are also included in the biodiversity subsidies, while representing a relatively small portion of the money (30 million CHF) allocated compared with subsidies allocated to “quality” and “network” surfaces (383 million CHF). Interestingly, for results-oriented quality 2 surfaces, local authorities (cantons) can edict some particular specifications to enhance the quality of biodiversity conservation locally. On top of the Q1/Q2 system, a third and partially overlapping category of subsidies is given to networks of valuable surfaces that allow regional projects. These complementary approaches, mixing top-down subsidies on quality surfaces and bottom-up support of local networks improving biodiversity, have been gradually put into force and adapted during the last three decades.

Switzerland is often considered a “green land” with 606,000 ha of pastures and natural meadows that altogether represent about 60% of the agriculture area (FOAG, 2022), pretty much in line with global standards (FAO, 2010). Forage crops mostly consist of various population mixtures of grasses (*Poaceae*) and legumes (*Fabaceae*). Important traits have been improved by breeding programs in some species, like yields, digestibility and disease resistance (Capstaff and Miller, 2018). In Switzerland, a list of optimised ecotypes is published annually and covers six legumes and sixteen grass species (Agroscope, 2023). All these species are also considered as priority CWR according to the recently published Swiss CWR checklist, except for *Onobrychis viciifolia* Scop. (Petitpierre et al., 2023). These ecotypes are usually sold and sown as mixtures. While a pro-active breeding effort is undergone for these forage crops, the long-term quantity and quality of forage produced by these meadows depend on their access to the largest possible genetic diversity. Across the country, there is a relatively large variety of meadows all over the various eco-geographical regions that are also cultivated very differently, from extensive to high-input intensive surfaces (Pazúr et al., 2021). Changes in management over time, particularly concerning tillage, fertilisation, early harvesting and over-sowing are important when aiming at conserving genetic diversity. One of the most significant examples is the estimated 98% decrease in *Arrhenatherion* meadows since the 1950s (Bosshard, 2015). Promoting ways to conserve the genetic diversity in these surfaces would also preserve the long-term security of an important gene pool for breeding in an ever-changing climate.

We describe here an innovative strategy to improve the conservation of the priority CWR of forage crops in Switzerland. We analyse the dynamics of deployment of the *in situ* program and try to evaluate the extent to which this program allows sufficient protection of both inter and intra-specific biodiversity. More generally, we discuss whether similar measures could be relevant for other low-intensive surfaces containing other CWR taxa. While new aspirations of the Global Biodiversity Framework have just emerged, developing new efficient strategies to manage plant conservation outside protected areas seems a very timely challenge.

## Description: designing the *in situ* program

The agri-environmental measures in Switzerland follow a cross-compliance scheme based on a complex combination of target and result-oriented measures. The focus on *in situ* conservation has been triggered by a political push included in the Swiss political agriculture plan 2014-17 (Swiss Federal Council, 2014), setting the base for novel measures that later coagulate into the *in situ* program. The objectives of the *in situ* program combine conservation of genetic diversity, sustainable use and long-term adaptation, pretty much in line with more general objectives like the Target 4 of the Global Biodiversity Framework (CBD, 2022). In this respect, the program wished to maintain the land-use intensity stable and without any addition of exogenous genetics over a relatively long period (at least eight years). Meanwhile, reporting of floristic quality and access to research and breeding is guaranteed.

The modalities of the *in situ* program have been designed by a working group gathering conservationists, scientists, breeders and representatives from local authorities and coordinated by the federal authorities. After an initial phase of design, three possible schemes could be short-listed, ranging from a standard top-down subsidy scheme to an entirely bottom-up network of saving/spending surfaces (**Table 1**). The first option allows identification by experts of particularly high-quality surfaces but was perceived as quite limited in its scope (low efficiency). It also struggled to comply with constitutional legal principles of “equal treatment” amongst beneficiaries. The bottom-up option (option 2, **Table 1**) was initially perceived as potentially heavy in terms of administrative burden but has been nonetheless deployed as an independent public-private initiative (Regioflora, 2023). Due to practical (more difficult seed harvesting) and financial (compensatory measures too expensive), this option was mostly concentrated over extensive surfaces. The third and only remaining option (auction-based, **Table 1**) that has been shifted into the pilot phase combined a trade-off between centralised quality control and bottom-up voluntary participation. Only surfaces were considered when (motivated) farmers actively participated in the published call in the first place.

**Table 1:** Three options were designed and discussed with the expert group before the implementation of the *in situ* program.

Options	Target	Recruitment type	Evaluation
1. Top-Down inventory	About 500 “good quality” surfaces (up to 10 surfaces for each of the 7 ecogeographical regions).	Top-Down (Experts)	Initially favoured but hard to implement (costs/benefits ratio too high). Legal issues over equality of chances among beneficiaries
2. Surface network	Establishment of donor surfaces network to ensure the “spread” of local genetics	Bottom-Up (Farmers)	This option has been developed in another non-legally binding mechanism and further tested and expanded
3. Auction-based scheme	Auction-based call for farmers to participate. Max target surfaces fixed.	Mixed	Less control over surfaces by the authorities but better compliance is expected

Option 3 has been retained for a secondary test phase in two pilot cantons (Graubunden and Luzern) for two seasons.

The *in situ* program offers to protect surfaces up to two hectares per farm in all ecogeographic zones. These surfaces must show a specific quality (no or neglectable neophytes and stable population for at least eight years without exogenic sowing) and correspond to one of the targeted species associations (**Table 2**). Importantly, these surfaces should not be already considered by any other agri-environmental measures and should be localised outside the grazing surfaces. This virtually tells that the *in situ* surfaces are a new space for conservation on agricultural land. Since its inception in 2021 and in the absence of *ex ante* quantifiable objective for a sufficient degree of conservation (see discussion below), a maximum quota of 2,750 hectares over the eleven Swiss biogeographic regions and 26 cantons has been set and opened for distribution. This quota also took into account sharing the administrative burden for every canton evenly. Eight plant associations were prioritised in total (**Table 2**) and at least one target taxa (**Table 3**) should be present on any surface considered in the program. The attribution scheme is one originality of the *in situ* program compared to the existing agri-environmental measures. Local public authorities (cantons) are regularly opening a call for surfaces until the quota of the maximum surface is attributed. The selected candidate surfaces are then checked, the floristic quality controlled by expert botanists and only the best surfaces retained in coordination with federal authorities. Noteworthy, the farmer is actually paying the controls him/herself: there is mechanically a first cost-benefits evaluation at the farm level that select for a particular subset of “motivated” participants. The selected surfaces are integrated into the program for the next eight years and receive a contribution accordingly (CHF 450.- per ha in 2022). To test the feasibility and relevance of the option 3 design, two pilots have been conducted starting in 2018 and 2019 in cantons Graubunden and Luzern respectively. Following a call organized by the local administrations, contributions have been distributed and the dynamics of adoption followed. These pilots allowed the identification of some limitations and issues in recruiting farmers who would be willing to participate. After some adaptations, the *in situ* program was deployed nationally in 2021 (with the first surfaces to be accounted for in 2022).

**Table 2:** List of the eight plant associations considered in the *in situ* program.

Name	Indicator taxa	Altitude	Intensity of cultivation	Ref.
*Arrhenatherion*	*Arrhenatherum elatus* *Campanula patula* *Crepis biennis* *Geranium pratense* *Malva moschata*	till 800 m	Semi-intensive	4.5.1
*Heracleum Dactylis*	*Dactylis* *Trifolium* *Taraxacum*	till 1200 m	Semi-intensive	11
*Lolietum multiflorae*	*Lolium multiflorum* *Poa trivialis*	till 600 m	Intensive	13
*Trifolio-alopecuretum*	*Alopecurus pratensis* *Trifolium, Poa, Dactylis*	till 1400 m	Semi-intensive	14
*Poo pratensis-lolietum perennis*	*Poa pratensis* *Lolium multiflorum* *Trifolium*	till 1400 m	Semi-intensive	15
*Polygono-trisetion*	*Campanula rhomboidalis* *Cardaminopsis helleri* *Centaurea pseudophrygia* *Muscari botroyides* *Narcissus radiiflorus* *Polygonum alpinum* *Thlaspi brachypetalum*	800-2000 m	Intensive	4.5.2
*Cynosurion*	*Crepis cappillaris* *Gaudinia fragilis* *Leontodon autumnalis* *Phleum bertolonii* *Senecio jacobaea* *Veronica filiformis*	till 1600 m	Semi-intensive	4.5.3
*Poion alpinae*	*Cerastium fontanum* *Crepis aurea* *Phleum rhaeticum*	1400-2500 m	Semi-intensive	4.5.4

Surface nomenclatures refer to two sources: Eggenberg et al., 2015 and Dietl and Jorquera, 2015. Indicator taxa are detrimental to identifying the association type but are mostly distinct from the primary target species of the *in situ* program (listed in **Table 3**).

**Table 3:** Target taxa of the *in situ* program and their representation on surfaces included in the program.

Target species	Number of surfaces
*Trifolium repens L. s.l.*	1029
*Dactylis glomerata L.*	931
*Lolium perenne L.*	838
*Poa pratensis L.*	704
*Trifolium pratense L. s.l.*	681
*Alopecurus pratensis L.*	503
*Trisetum flavescens (L.) P. Beauv.*	469
*Festuca rubra aggr.*	362
*Lolium multiflorum Lam.*	332
*Arrhenatherum elatius (L.) J. & C. Presl*	329
*Festuca pratensis Huds. s.l*	282
*Cynosurus cristatus L.*	210
*Phleum pratense L.*	148
*Lotus corniculatus L.*	88
*Festuca arundinacea Schreb. s.l.*	78
*Agrostis gigantea Roth*	29
*Medicago sativa L.*	19
*Onobrychis viciifolia Scop.*	9

At least one of these taxa must be present on the surface to pretend to be included in the *in situ* program. Noteworthy, all taxa have been considered as Swiss priority CWR except for *Onobrychis viciifolia* (Petitpierre et al., 2023).

## Methods

Each site included in the *in situ* program is characterised by spatial coordinates, area, abundance of the target species and attributed association. The identification of plant associations was done by the farmers upon subscription and verified by certified botanists during the selection process, as described in the previous paragraph.

### Potential distribution of the sites and stratified sampling

We analyzed the disparity between the actual distribution of sites from this bottom-up *in situ* program and a stratified sampling across environmental and geographical gradients. Such balanced sampling is anticipated to catch a broad spectrum of the intra-specific genetic diversity (Tobón-Niedfeldt et al., 2022). For this objective, it was essential to determine the potential distribution of the candidate meadows throughout Switzerland’s biogeographical regions and agricultural zones. To map out the complete potential distribution of the candidate sites within the program, we used the shapefiles of the agricultural area in Switzerland (FOAG, 2023). We retained only areas corresponding to permanent meadows, pastures and wooded pastures *i.e.* areas with codes 613, 616 and 625. These categories are the only eligible for the *in situ* program. Moreover, we excluded ECAs from the layers as they belong to other subsidy instruments (**Supplementary Figure S1**).

The GIS layers were sourced from the Federal Office of Agriculture in April 2023. For faster data manipulation, vector layers were rasterized to a 10 m resolution using the *terra* library (Hijmans et al., 2023) in the R software, version 4.0.3 (R Core Team, 2020).

To delineate different strata along biogeographical and altitudinal gradients, we overlaid the map of Switzerland’s biogeographical regions (FOEN, 2022) with the map of its agricultural zones (FOAG, 2023). Biogeographical regions are delineated based on shared similar ecological characteristics and history. However, they lack a finer subdivision along the altitudinal gradient (FOEN, 2022). For this, the distinct agricultural zones of Switzerland are useful as they are subdivided based on altitude levels, climate, transport routes and topography. They encompass seven zones, ranging from the lowlands to the alpine levels (FOAG, 2023). Out of 84 theoretical units (12 bioregions * 7 agricultural zones), we identified 66 biogeographical strata, as some of the strata were not present across all the biogeographical regions (**Supplementary Figure S2**).

Therefore, we could identify the potential area for the *in situ* program in each stratum (**Supplementary Table 1**). Balanced stratified sampling assumes an equal sampling effort within each stratum. However, some strata have only very few potential areas (**Supplementary Table 1**). For strata with a potential area equal to or lower than 420 ha, we assumed that only one-tenth of these small areas could be sampled. Therefore, a proportional sampling was assessed in these small strata.

### Potential distribution of the associations

For each association, the list of characteristic, dominant or companion species was sourced from Dietl and Jorquera, 2015; Eggenberg et al., 2015 (**Supplementary Table 2**). For each of these species, a potential distribution map was derived from species distribution models (SDMs) linking species observations to topo-climatic layers (Guisan and Thuiller, 2005; Elith and Leathwick, 2009). We used the same methods, data and environmental datasets as described in Petitpierre et al., 2023. To summarize, species with enough occurrences (n ≥ 10) were associated with an initial set of 33 environmental layers describing topography, climate and remote sensing factors. An initial selection process retained only the most relevant variable, keeping between two and nine variables, depending on the species (see Appendix 1 in Petitpierre et al. (2023)). These variables were then related to species occurrences by combining three modelling algorithms (general additive models, MaxEnt and gradient boosting model) into an ensemble modelling approach (Thuiller et al., 2004; Araujo and New, 2007), or an ensemble of small models (ESM, Breiner et al., 2015) depending on the number of observations. This approach produced continuous suitability maps for each species at a resolution of 100 m. These continuous maps were reclassified into binary maps (*i.e.* potential presences and absences) by setting a suitability threshold to obtain an omission ratio of 10%. The map of the potential distribution of each association was then obtained by stacking the potential distribution maps of the species contained in the association. In the stacking, maps of characteristic species were given a weight of 1.2 while the other species had an assigned weight of 0.2, *i.e.* five times less than the characteristic species. Therefore, the stacking of the species potential maps of each association produced a map of a continuous association score. This score was also reclassified into a binary score (*i.e.* favourable or unfavourable) by comparing the potential distribution of the association with the observed distribution of the associations in the InfoFlora database (Infoflora, 2023), which is the competence centre for information on the wild plants of Switzerland. It contains more than 11’000’000 observations of the Swiss flora and associations score were calculated at a 100 m resolution for Switzerland. The continuous association score of the potential maps of the associations was binarized by setting a threshold to obtain an omission ratio of 5% regarding the observed distribution of the associations, except for *Poion alpinae* where it was set to 7.5% for a more plausible distribution, according expert’s opinion.

### Priority CWR beyond the primary target species of the *in situ* program

To obtain a more exhaustive list of plant species located at each site of the *in situ* program (**Supplementary Table 3**), we extracted the observations from the InfoFlora database in a radius of 75 m around the coordinates of each site and kept the observations of the priority CWR of Switzerland. Only occurrences observed between 01.01.2002 and 31.08.2022, and with a coordinate uncertainty smaller than 150 m were retained. Importantly, as the observations of the InfoFlora database consist of different types of datasets (opportunistic observations, monitoring, surveys), more species can be present around the sites and not being reported in the database.

## Impact of the *in situ* program on forage crop diversity

### Adoption rates and actual proportion of surfaces incorporated

**Figure 1:**
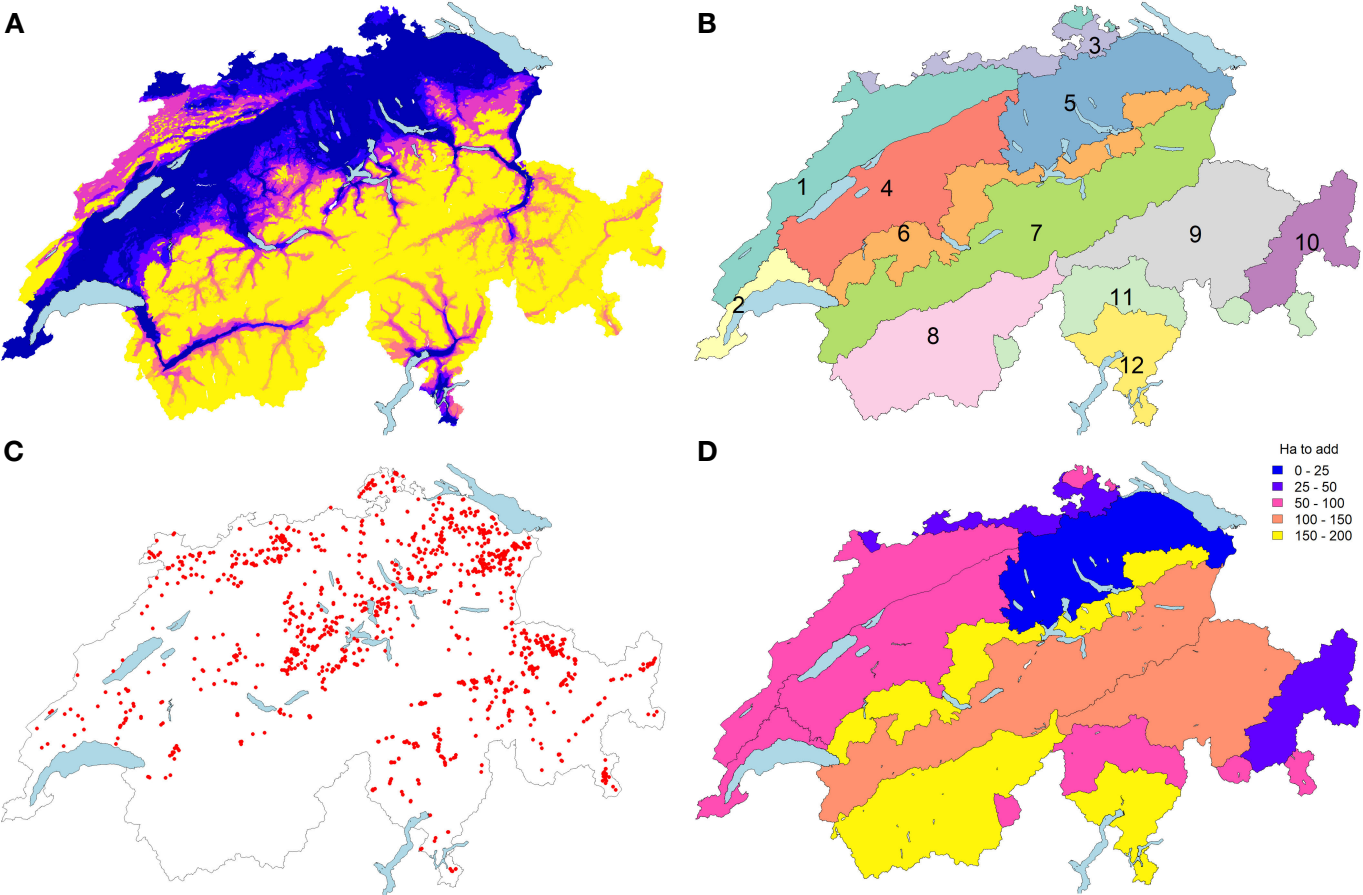
The seven agricultural zones **(A)** were crossed with 12 bioregions **(B)** to delineate 66 biogeographic units in Switzerland. The current distribution of surfaces subscribing to the *in situ* program **(C)** is contrasted with theoretical targeted needs to get a distribution approaching a balanced stratified sampling of 2750 ha **(D)**. Bioregions of Switzerland consist of Jura and Randen (1), Lake Geneva Basin (2), Rhine Basin (3), Western Plateau (4), Eastern Plateau (5), Prealps (6), Northern Alps (7), Western Central Alps (8), Eastern Central Alps (9), Engadin (10), Southern Alps (11) and Southern Ticino (12).

Taking the actual stand of deployment (57% quotes filled), the next point is, therefore, to identify where the next surfaces should be ideally situated to significantly improve the conservation of target species and associations. To what extent would the 1’566 hectares included in the program provide sufficient coverage? And if not, how to incentivize the other cantons or farmers to participate? Is the auction-based system the more appropriate scheme when the offer is not sufficient to fill the quotas? Two years after inception, 1’217 surfaces could be integrated into the *in situ* program, covering a total of 1’566 hectares (**Figure 1C**). This covers 57% of the allocated quota (2’750 ha) distributed in 23 out of 26 cantons. Some cantons simply did not find any suitable surfaces in the first round, other were reluctant or even refused to participate, as the program was judged not worthy in terms of costs/benefits balance. While the modalities of the *in situ* program are by definition bottom-up, there is no direct control over the localization of the surfaces being integrated, nor their floristic quality. The relative quantity of each association within the allocated surfaces is only monitored indirectly.

### Evaluation of sampling needs and potential biases

Considering the surfaces already integrated into the program (**Figure 1C**), the sites are distributed across 49 biogeographical strata (out of 66; 75%). The potential area for the eligible sites is bigger than 100 ha in 9 empty strata (**Supplementary Table 1**). We can observe a bias in surfaces considered in the *in situ* program towards the East of the country (**Figure 1**). A balanced stratified sampling of 2’750 ha would tend towards 51.63 ha in each stratum with a potential area ≥ 420 ha and 10% of the potential area in strata with a smaller potential (surfaces for each biogeographical unit (**Figures 1A, B**; **Supplementary Table 1**). The current distribution of the sites is only weakly correlated to such a balanced stratified sampling (Pearson’s correlation = 0.33, p-value = 0.007) with a significantly different distribution (Chi-square test p-values <0.001). To reach a distribution close to a stratified sampling, the next sites should in priority target the Prealps, Central Western Alps and Southern Ticino regions with about 150 to 190 ha to add, while Rhine Basin, East Central Alps are slightly over-represented with an excess of about 120 ha (**Supplementary Table 1**). It appears that the biogeographical pattern of the deficit in surfaces occurs mostly in the Alps (**Figure 1**), but when looking at the finest strata level, lowlands in South-Western Switzerland should also be complemented to tend to a more balanced distribution across the strata (**Supplementary Figure S3**). This analysis also reveals that Engadin, Eastern Plateau and the Rhine basin are already well covered by the existing network of surfaces, with only 15 to 50 ha to add in these regions (**Supplementary Table 1**, **Supplementary Figure S2**). The current distribution of the surfaces is correlated to the distribution of a proportional sampling, *i.e.* to the distribution of the potential candidate meadows (Pearson’s correlation = 0.81, p-value <0.001) but remains significantly different (Chi-square test p-values <0.001, **Supplementary Table 1**).

### Representation of plant associations

**Figure 2:**
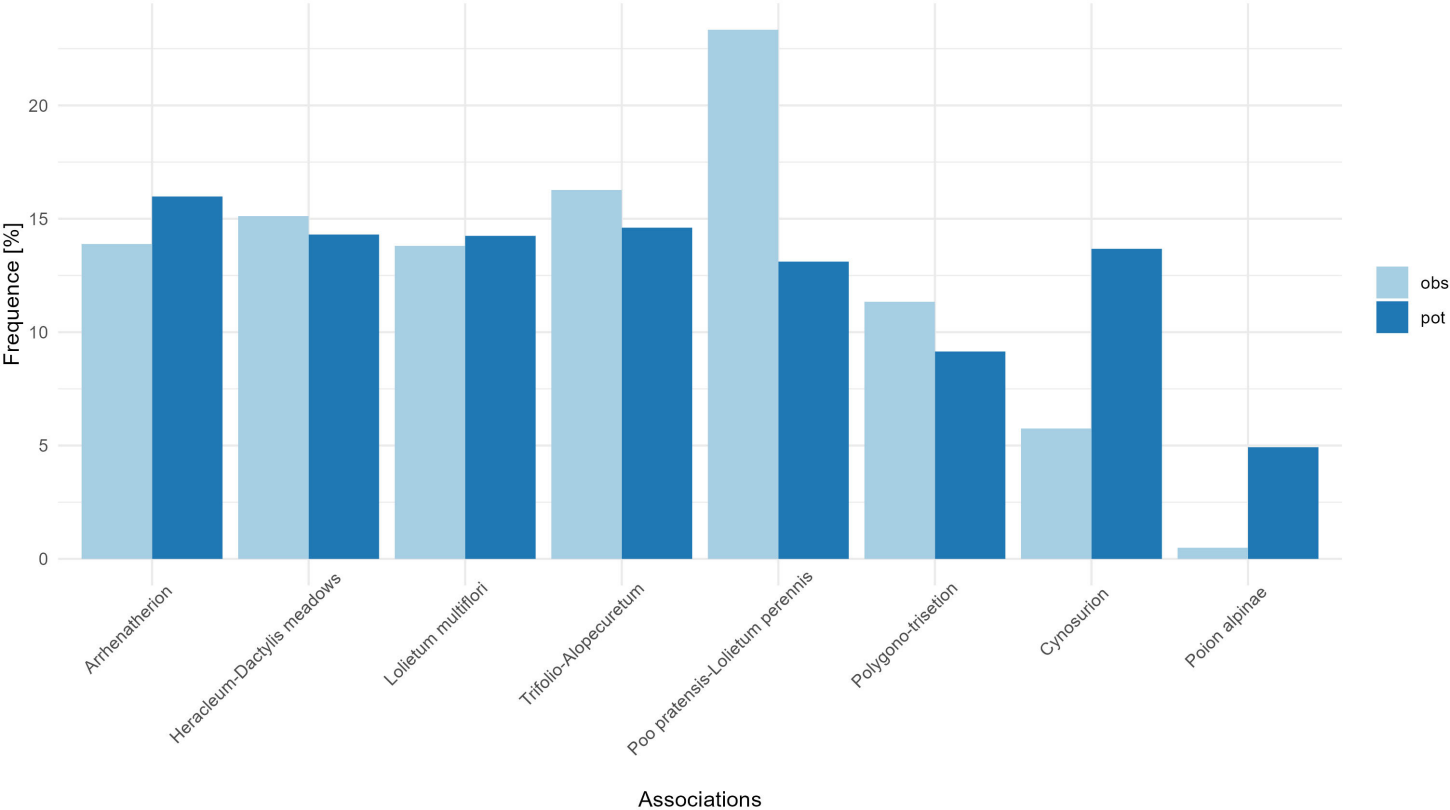
Frequency of each plant association among the current sites of the program (light blue, obs) and the modelled distributions of these associations (dark blue, pot). Detailed composition of each plant association is given in **Table 2**.

Next, based on floristic data controls, we assessed the actual proportions of each plant association in the instrument after two years of inception (**Figure 2**). To check whether the frequencies of the associations that have been included in the *in situ* program are representative, we compared the current observed distributions with the modelled distributions of each association. The associations’ frequencies of the current surfaces are positively correlated with the associations’ potential distribution frequencies (Pearson’s correlation = 0.64) but this correlation is not significant (p-value = 0.086, **Figure 2**). A Chi-square supports that the current frequency of the associations is independent of the frequency of the modelled distributions of the associations (p-value = 0.016). This indicates a relatively even representativity of each association except for the overrepresentation of *Poo pratensis-lolietum perennis* and the underrepresentation of *Cynosurion* and *Poion alpinae* (**Figure 2**). While *Poion alpinae* might not be the most threatened association considered in this program, its floristic quality is highly dependent on nitrogen levels/intensification and therefore very unevenly distributed (Eggenberg et al., 2015). It is also possible that many *Poion alpinae* surfaces are already integrated into other instruments dedicated to pastures, and therefore not considered here.

**Figure 3:**
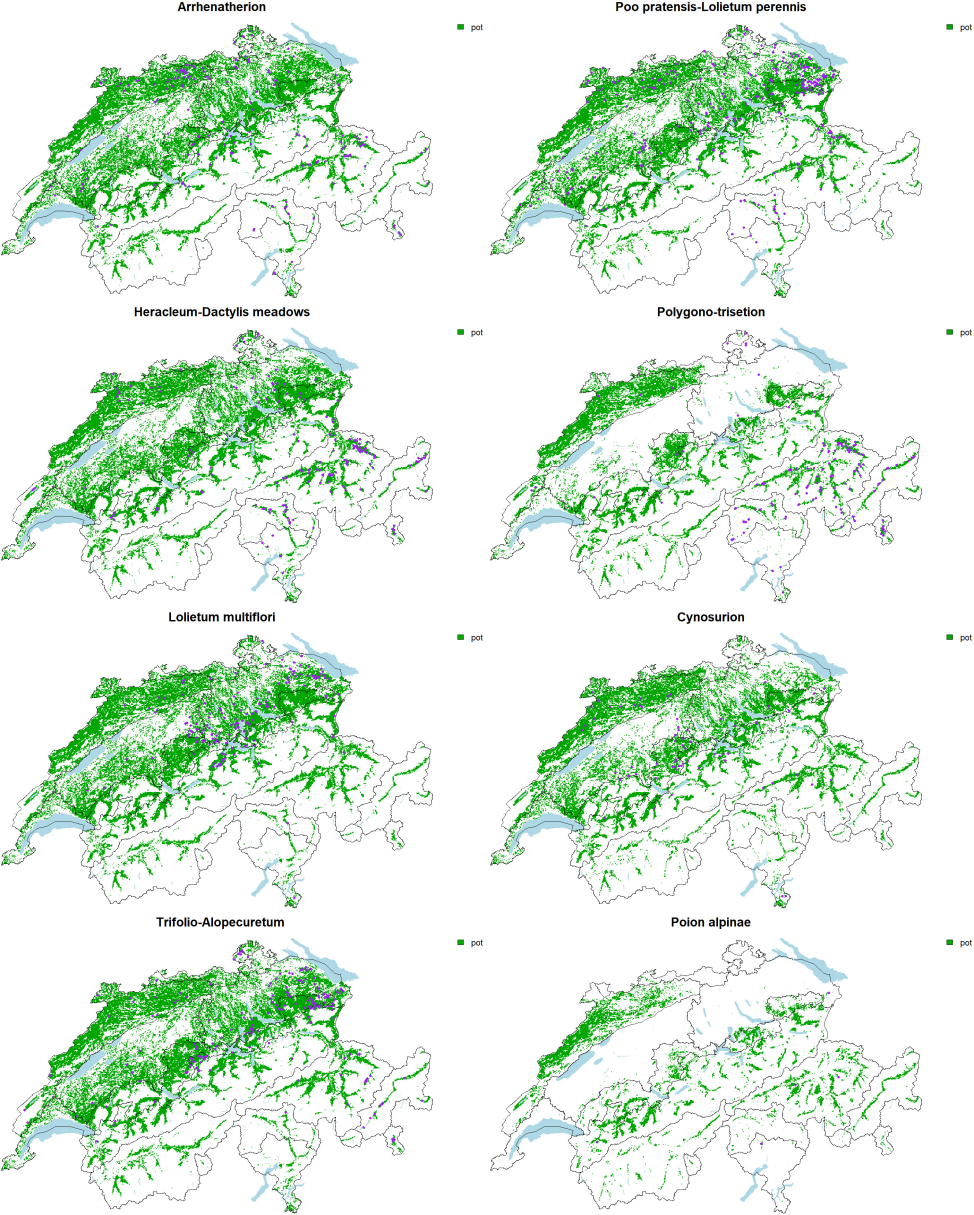
Potential suitability for the eight associations (green area, pot) and current sites of the program belonging to the association (purple dots).

To refine the evaluation of the effectiveness of the *in situ* program, and to allow a diagnostic on whether and how to fill the rest of the surface quotas, we evaluate for each association their potential suitability and compare it to the actual surfaces already in the instrument. Visually comparing the suitability of each association to the actual extent of surfaces in the program allows a quick and easy diagnostic (**Figure 3**). Typically, while a vast majority of the *Arrhenatherion* surfaces are located in the Jura and Eastern Switzerland, the suitability map flags other potentially interesting surfaces in the Western Plateau and the Western Central Alps (**Figure 3**). *Arrhenatherion* representation in the program is almost comprehensive compared to the frequency of other associations (**Figure 2**). However, more sampling effort is necessary in these underrepresented regions to cover the diversity across the full elevational and biogeographical gradient.

### Impact of the *in situ* program on CWR

*Primary targets.* All 18 target species are logically distributed across the 1’217 current sites included in the *in situ* program. However, observation data were missing for 5 sites in the analysed dataset. The rarest species is *Onobrychis viciifolia* (9 sites), whereas *Trifolium repens* is the most widely distributed, observed in 1’029 sites (**Table 3**). On average, each target species can be found in 391.17 +/- 326.86 sites. Each site includes between 1 and 15 target species, with an average of 5.79+/- 2.12 target species.

*Other Priority CWR.* The presence of at least one of the 18 target species is mandatory to qualify a surface for the *in situ* program. Except for *Onobrychis viciifolia*, these species are listed as national priority CWR. Meanwhile, the extent to which other priority CWR from the 285 taxa of the Swiss priority CWR checklist (Petitpierre et al., 2023) could also be present in these surfaces remains unexplored. These “secondary” targets could also be considered contributing to increase the actual effectiveness of this instrument. Other Swiss priority CWR were found in 358 sites (29%). These sites comprise between 1 and 18 supplementary priority CWR. In total, the current distribution of the sites includes 108 priority CWR (38% of the taxa; **Supplementary Table 3**). There are between 1 and 24 priority CWR per site, with an average of 6.76 +/- 2.88 priority CWR species per site. 9 sites (0.74%) comprise 8 species with a threatened Red List status (i.e. CR, DD, EN, NT, RE or VU). The contribution of the *in situ* surfaces to the overall diversity conservation appears therefore positive, not only for the primary targets but also for other priority CWR.

## Actionable recommendations

### Considering various diversity levels for an efficient CWR conservation

Most CWR conservation strategies focus on the “taxa” level (Maxted et al., 2006), but conserving biodiversity is more complex. Taking advantage of the example of *in situ* conservation of forage plants presented here, we would like to emphasize the various aspects that need to be considered for an efficient conservation design, namely specific and ecosystem diversity (taxa/species numbers and plant populations), phylogenetic diversity (particularly between crops and CWR) and finally intra-specific or allelic diversity.

#### Specific diversity

The most accessible “level” of diversity in plants is surely through the taxa representation, but care should be taken to also consider the diversity between communities and at landscape scale (referred to as beta and gamma biodiversity respectively). Agricultural practices using forage plants offer an interesting example of the importance of plant associations: identification and work at the farm level do indeed exclusively occur at the association level (and not at the taxon level). In Switzerland, conventional breeding work is concentrated at the single-taxa level, while the products of these forage crops are subsequently commercialized as mixtures (aiming at rebuilding the various plant associations). The primary targets of the *in situ* program were therefore gathered in 8 distinct plant associations (**Table 3**), but it is important to consider that differences among the associations themselves can be relatively important (depending for example on the level of fertilisation). Some meadow restoration programs use local seed transfers without systematically considering the actual identity or genetic diversity of the taxa transferred (Hedberg and Kotowski, 2010; Listl et al., 2018; Slodowicz et al., 2023). These practices risk the uniformization of the genepool in the long term. The *in situ* program uses plant association and their relative distribution as a proxy to conserve the beta biodiversity, while not explicitly measuring it.

#### Phylogenetic diversity

In addition, the inter-taxon relationship (the phylogenetic diversity) can also be considered to evaluate the diversity of target surfaces (Laity et al., 2015; Gumbs et al., 2021; Gonzalez-Orozco and Parra-Quijano, 2023). Considering phylogenetic diversity is particularly important to the CWR context, as the phylogenetic distance from a CWR to its crop is often linked to its actual use in breeding programs. To emphasize the importance of cytogenetic compatibility, some authors refer to crop wild phytorelatives (CWP, Viruel et al., 2021) to better illustrate this nuance and help in possible prioritizing. More work is necessary to integrate phylogenetic diversity into the *in situ* program and try to understand the dynamic evolution of the meadow’s population.

#### Intra-specific/allelic diversity

Intra-specific genetic diversity of forage grasslands, i.e. diversity below the taxonomic species level, is rarely considered and its measure is relatively demanding (Flanagan et al., 2018). However, allelic diversity appears as a key element for effectively informing conservation planning. For example, using multispecies amplicon sequencing allowed a primary evaluation of the allelic diversity of some meadows for grasses and legumes species (Loera-Sanchez et al., 2022). Such approaches may be useful for the *in situ* program: they can bring more insights into the genetic variability gathered by this instrument and set possible ways to control its evolution through time. A spatially stratified approach to population selection for an *in situ* conservation strategy has been shown to improve allelic conservation and intra-specific diversity (Tobón-Niedfeldt et al., 2022). Such a quantifiable measure of efficiency of the *in situ* program could in turn allow better tailoring of the measures and complementary surfaces.

The *in situ* program presented here takes into consideration the specific diversity, both at a taxa and association level, but data are lacking for both phylogenetic and intra-specific/allelic diversity. The underlying rationale that “more surfaces are better”, which has essentially been driven by practical or political reasons remains to be shown and a lot of questions remain open: what are the optimal surfaces to achieve decent conservation of priority CWR (is 2’750 ha a reasonable target)?, and to what extent the de facto fragmentation of the measure is not a limiting factor to achieve the conservation objectives, particularly in terms of allelic and phylogenetic diversity? At best, our analysis shows that the sites allocated so far do not completely cover altitudinal and biogeographical gradients, whereas stratified sampling along these gradients is crucial for preserving overall diversity (e.g. Gonzalez-Orozco and Parra-Quijano, 2023). More research included in the monitoring and evaluation of the *in situ* conservation program is needed to allow a clearer evaluation of the efficiency of conservation of the priority CWR through these instruments. For example, there is not enough data to formally determine whether there are enough surfaces (planned or effectively deployed) to conserve rare allelic variations (Whitlock et al., 2016).

Considering its moderate cost and fast inception speed, if proven successful in maintaining diversity, the *in situ* program could be extended to other priority CWR: 285 taxa have been prioritised in a national survey, of which 18 are included in the actual *in situ* programs (Petitpierre et al., 2023). The extension of the *in situ* program to other CWR will take advantage of the 39 complementary CWR regions that have been identified during the survey and will consider CWR of food, feed and medicinal crops (Petitpierre et al., 2023).

Most *in situ* conservation policies are led side by side with *ex situ* conservation (e.g. Phillips et al., 2016). Informing and coordinating the *in situ* conservation of CWR while flagging the need for *ex situ* conservation appears like a reasonable approach. It is referred to as the trans situ conservation (Riordan and Nabhan, 2019). Trans situ conservation aims primarily at coordinating in and *ex situ* conservation efforts possibly in a single coherent policy effort, to improve the efficiency of conservation, avoid duplication of work and modulate possible storage artefacts like genetic drift (Lauterbach et al., 2012). In Switzerland, *ex situ* conservation of wild plants and in particular crop wild relatives is still in its infancy. Standardized protocols are missing, and the *ex situ* conservation of complex seed mixtures from priority populations would require more research. As described for the *in situ* program, eco-geographical distribution modelling can be very informative for the efficient design of *ex situ* conservation policy as well. Generally, fluctuating and contradictory standards for *ex* and *in situ* conservation would greatly benefit a stratified and more systematic approach.

### *In situ* conservation of CWR as a policy challenge

The global governance of plant genetic resources historically first concentrated on *ex situ* conservation (Curry, 2022). Following a recent paradigm shift, it is now also considering *in situ* conservation as a critical approach to conserving biodiversity. In its second report on the State of the World plant genetic resources, the FAO wrote: “*There is a need for more effective policies, legislation and regulations governing the on-farm management of plant genetic resources for food and agriculture, both inside and outside protected areas*” (FAO, 2010). This view is combined with the Seed Treaty provision on the conservation of CWR in its Art. 5d: “*Promote in situ conservation of wild crop relatives and wild plants for food production, including in protected areas, by supporting, inter alia, the efforts of indigenous and local communities*” (FAO, 2010) and provide strong legal support to CWR *in situ* conservation. Several examples of *in situ* measures have already been reported, for example, the four types of *in situ* CWR measures spreading across more than 57 initiatives across Europe: CWR genetic networks, potential genetic networks, people and institutional networks and project-associated initiatives (Alvarez Muniz et al., 2020). It also appears that a vast portion of so-called *in situ* conservation happens passively, *i.e.* including CWR already included in an existing protected area. Surveys on *in situ* measures for CWR reveal a large array of practices and tend to overestimate the actual measures dedicated to CWR conservation (Rubio Teso et al., 2021). The CWR conservation, particularly for taxa that are not necessarily threatened or are situated on agricultural land (Petitpierre et al., 2023), does fall in a “grey zone” between conventional conservation (red-list based) strategies and *ex situ* collections for breeding purposes. The *in situ* program is an attempt to address this conservation gap.

One of the first challenges to answer to the requirements of the international framework is to build measures that would be adapted to the Swiss context, particularly to its complex federal structure and subsidies regime. After an extensive consultation and testing phase, the current model inception combined a top-down resource allocation covering up to a theoretical maximum 2’750 ha and a bottom-up approach, where farmers could decide whether to participate in the program. Now two seasons into the program, half of the surface’s quota has been assigned. Two major issues appeared: 1. The addition of a new -supplementary- instrument on top of the whole subsidy machinery is a clear disincentive for farmers. While the instrument concentrates on new surfaces not yet considered by other measures, it remains that the cost-benefit for participation might be perceived as too high. Integration of the *in situ* program into other existing subsidies or cross-compliance programs might be a possible alternative. Overall, participation in the *in situ* program was strongly correlated with the support and dedication of the local authorities. 2. The bottom-up approach does not allow for fine control over the surfaces to be targeted: it is not necessarily the best or enough surfaces that will be integrated at first (like the *Poion alpinae* deficit observed in the current state of deployment, **Figure 2**). A complementary approach that would specifically target complementary high-value populations could be a possible way forward. Some additional target associations could also possibly extend the scope of the instrument, like for example the acidophilic *Festuca-Agrostis* meadows. In any case, the involvement of local stakeholders and experts in the flagging of high-value surfaces would be necessary. However, favouring certain farmers over the quality of their surfaces could breach some legal requirements related to equal treatment.

The *in situ* program represents about 1% of the total amount allocated to biodiversity subsidies to agrobiodiversity and a very small fraction of the surfaces integrated in these schemes (representing in total 190’609 ha in 2022, (FOAG, 2022). Meanwhile, extensive meadows and pastures still represent the vast majority targeted by ECAs. Therefore, it may be important to consider the overlaps between *in situ* surfaces and other ECA surfaces in terms of plant associations, (given that one condition to add a surface to the *in situ* program is that they should not be covered by other ECAs) it remains possible that some species or populations would be already under the scope of other measures, for example as Q1 or Q2 surfaces. Identifying such overlap is essential to avoid redundancy and guarantee overall policy coherence. In addition, as with any wild species, the CWR taxa considered in the *in situ* program are generally also present outside the agricultural area, and the distribution and diversity of the population outside cultivated meadows should also be taken into account for conservation. In such cases, species-rich surfaces -may- be protected under the various networks of natural reserves and other protected areas. Meanwhile, 22% of Swiss priority CWR were not significantly better covered by protected areas (excluding the *in situ* surfaces) than any random species (Petitpierre et al., 2023). Taken together, the exact relationship between *in situ* surfaces as a relatively restricted portion of the overall grass (cultivated or natural) meadows emphasizes the complexity of evaluating the instrument, particularly at the taxa level.

Although it appears in our analysis that considering conservation of forage CWR at the population level can in practice be a promising way to mobilize resources at the farm level. An additional concern towards the efficiency of the instrument that cannot be disregarded is the timing of obligations. A minimal 8-year engagement was foreseen in the *in situ* program, with controls being held regularly. While 8 years is a significant time for a farmer, it remains a possible weakness that can hardly be addressed: It is hoped that once the first round is over, the political and farmer commitments will not be eroded. Long-term management (particularly along altitudinal gradients) should take into consideration climate change. Additional work is needed to evaluate the extent to which the *in situ* surface network will be impacted, and whether measures could be already taken to mitigate these changes. Several modelling have already been performed on local case studies, like in Holland (Treuren et al., 2017) and Norway (Phillips et al., 2017) and include recommendations towards better coordination with *ex situ* conservation.

## Conclusion

Conservation strategies need to be dynamic and adapt to changes in the natural processes involved and the many anthropogenic cues that are altering habitats (Pressey et al., 2007). Particularly for *in situ* conservation, this is a timely challenge, given that European grassland alpine ecosystems are predicted to be heavily impacted by climate change (Schwager and Berg, 2019). The *in situ* program is a novel way of thinking about how to extend conservation outside protected areas towards agricultural lands. Other initiatives that aim to empower local communities and farmers in the conservation of CWR are also emerging gradually (Wainwright et al., 2019; Drucker et al., 2023). This is in line with the newly agreed Global Biodiversity Framework and the consideration of “other efficient conservation measures” (OECM) that are key elements of Target 2 of the GBF (IUCN WCPA Task Force on OECMs, 2019; CBD, 2022). One of the biggest advantages and simultaneously challenges of the program is the bottom-up design, based on a voluntary subscription by farmers themselves. Interestingly, it appears that most difficulties in the program onset were originating from local administrative bodies rather than farmers. This approach fosters a high degree of compliance and motivation: on one hand, only farmers who were concerned and motivated participate: It secures an element of recognition in the broader role of farmers towards agroecosystems and society at large. On the other hand, it shows some limits when it comes to comprehensively safeguarding biodiversity along the whole altitudinal and biogeographical gradients. It seems essential to use complementary conservation programs to target complementary valuable areas that would not have been successfully covered by such an auction-based program. Beyond the Swiss case study, the *in situ* program could represent a very promising strategy to improve CWR conservation and allow to protection of agrobiodiversity at large.

## Author’s note

The views and opinions expressed in this article are those of the authors and do not necessarily reflect the official policies or positions of the FOAG or InfoFlora.

## Author contributions

CK: Conceptualization, Data curation, Formal Analysis, Validation, Writing – review & editing. BP: Conceptualization, Data curation, Formal Analysis, Investigation, Methodology, Software, Supervision, Validation, Visualization, Writing – original draft, Writing – review & editing. PM: Validation, Writing – review & editing. YL: Conceptualization, Writing – review & editing. SE: Formal Analysis, Investigation, Validation, Writing – review & editing, Supervision. SA: Conceptualization, Funding acquisition, Investigation, Methodology, Project administration, Supervision, Writing – original draft, Writing – review & editing, Formal Analysis, Validation.

## Supplementary material

Supplementary Table 1 Distribution of the potential area and the number of sites across the biogeographical strata.

Supplementary Table 2 Species composition of each plant associations (characteristic and dominant species are distinguished).

Supplementary Table 3 Additional priority CWR covered by the current sites of the *in situ* program to the primarily targeted taxa.

Supplementary Figure 1 Potential distribution of the eligible meadows for the *in situ* program (green area). It corresponds to the agricultural area classified as permanent meadows, pastures and wooded pastures (i.e. areas with codes 613, 616 and 625). Ecological compensation areas were removed since they are part of other subsidy instruments. The original 10 m resolution raster was aggregated at a 500 m resolution for better readability.

Supplementary Figure 2 Distribution of the biogeographical strata in Switzerland

Supplementary Figure 3 Theoretical targeted needs at the strata level to get a distribution approaching a balanced stratified sampling over 2’750 ha.
