# Supplementary figures and images for: Fostering in situ conservation of wild relatives of forage crops
Source: Front Plant Sci. 2023 Oct 30;14:1287430. doi: 10.3389/fpls.2023.1287430 (PMC10643147; doi:10.3389/fpls.2023.1287430)

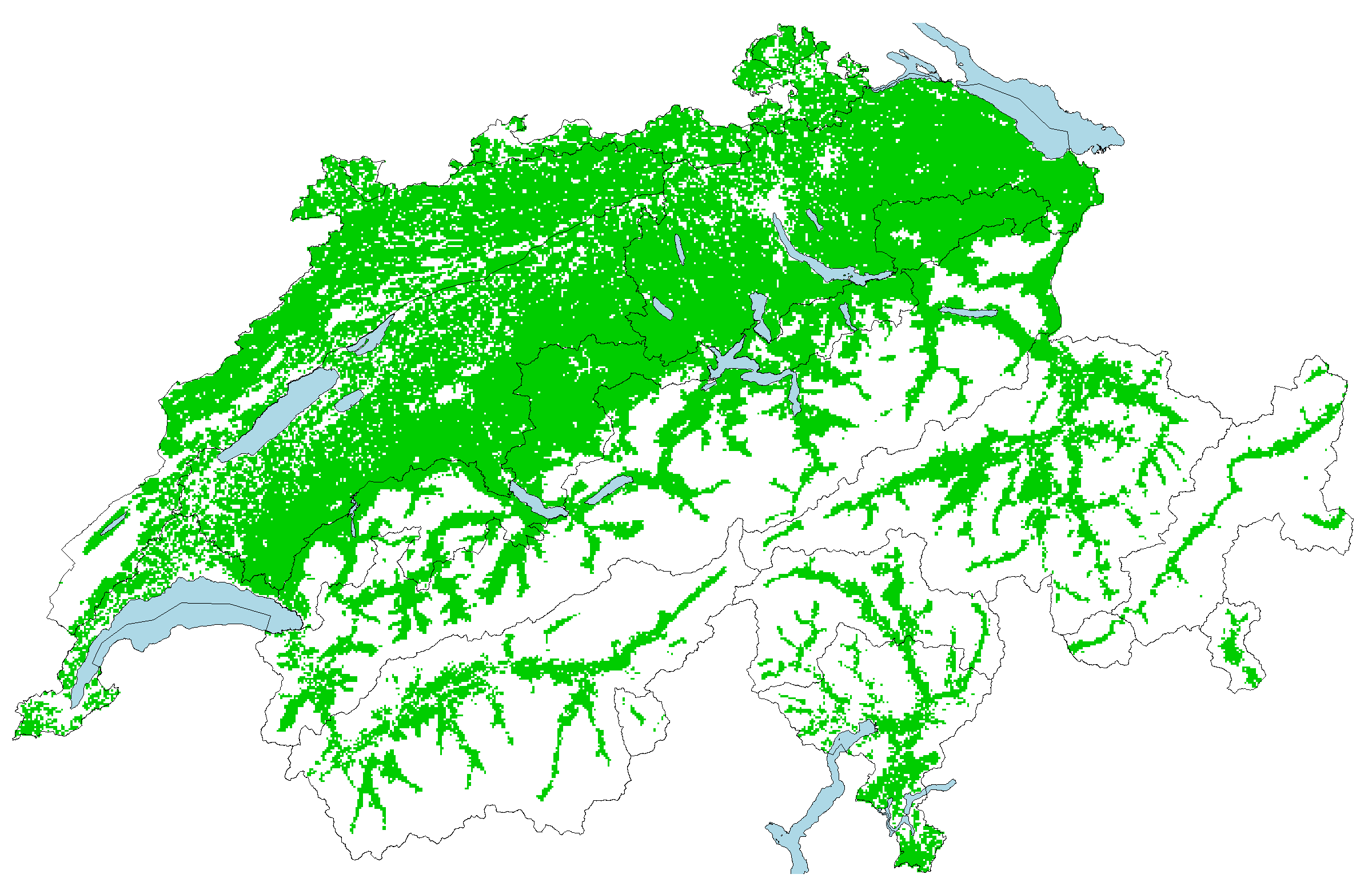

Supplement: Supplementary Figure 1 — Potential distribution of the eligible meadows for the in situ program (green area). It corresponds to the agricultural area classified as permanent meadows, pastures and wooded pastures (i.e. areas with codes 613, 616 and 625). Ecological compensation areas were removed since they are part of other subsidy instruments. The original 10 m resolution raster was aggregated at a 500 m resolution for better readability. [file Image_1.png]

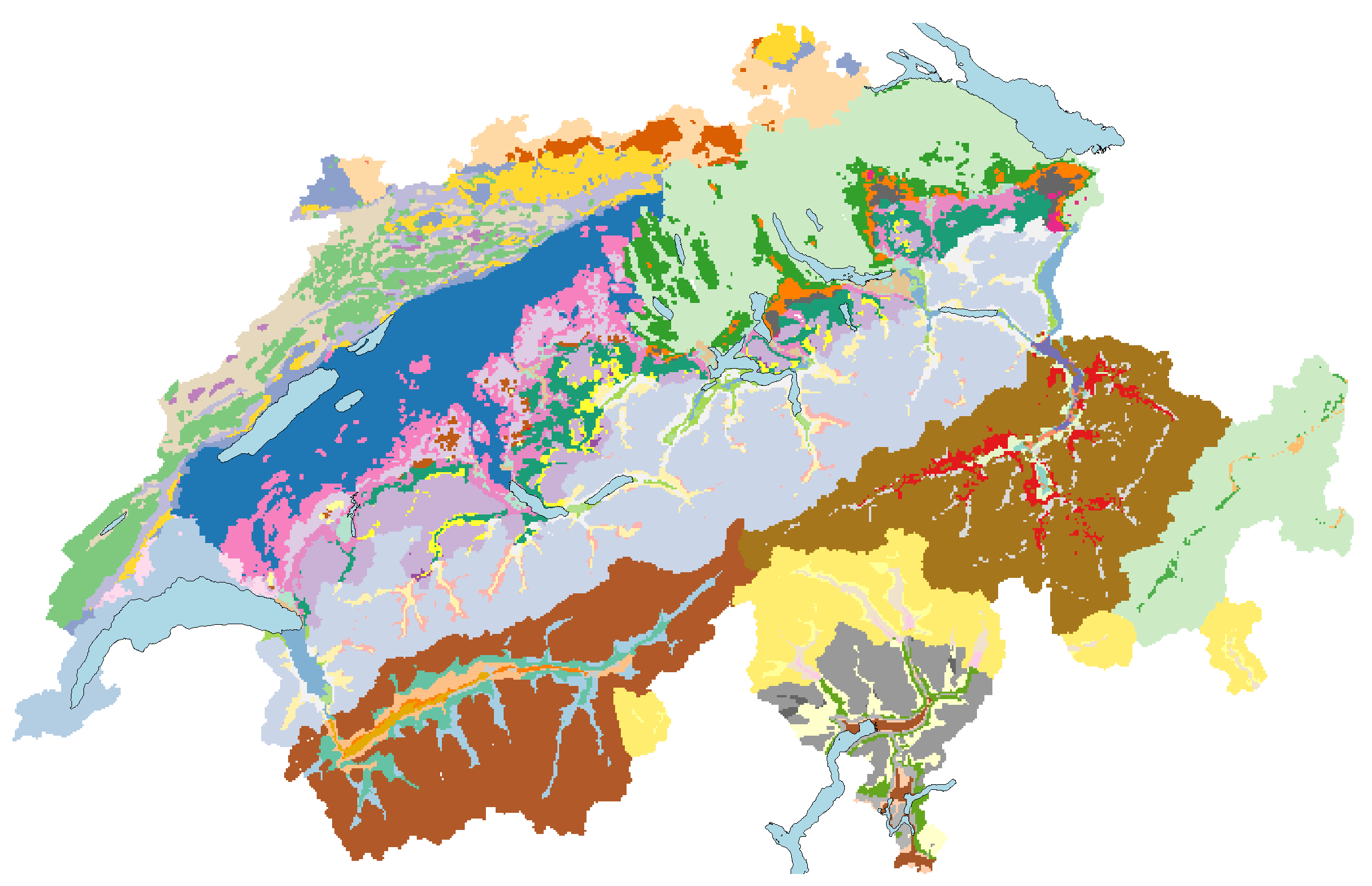

Supplement: Supplementary Figure 2 — Distribution of the biogeographical strata in Switzerland [file Image_2.png]

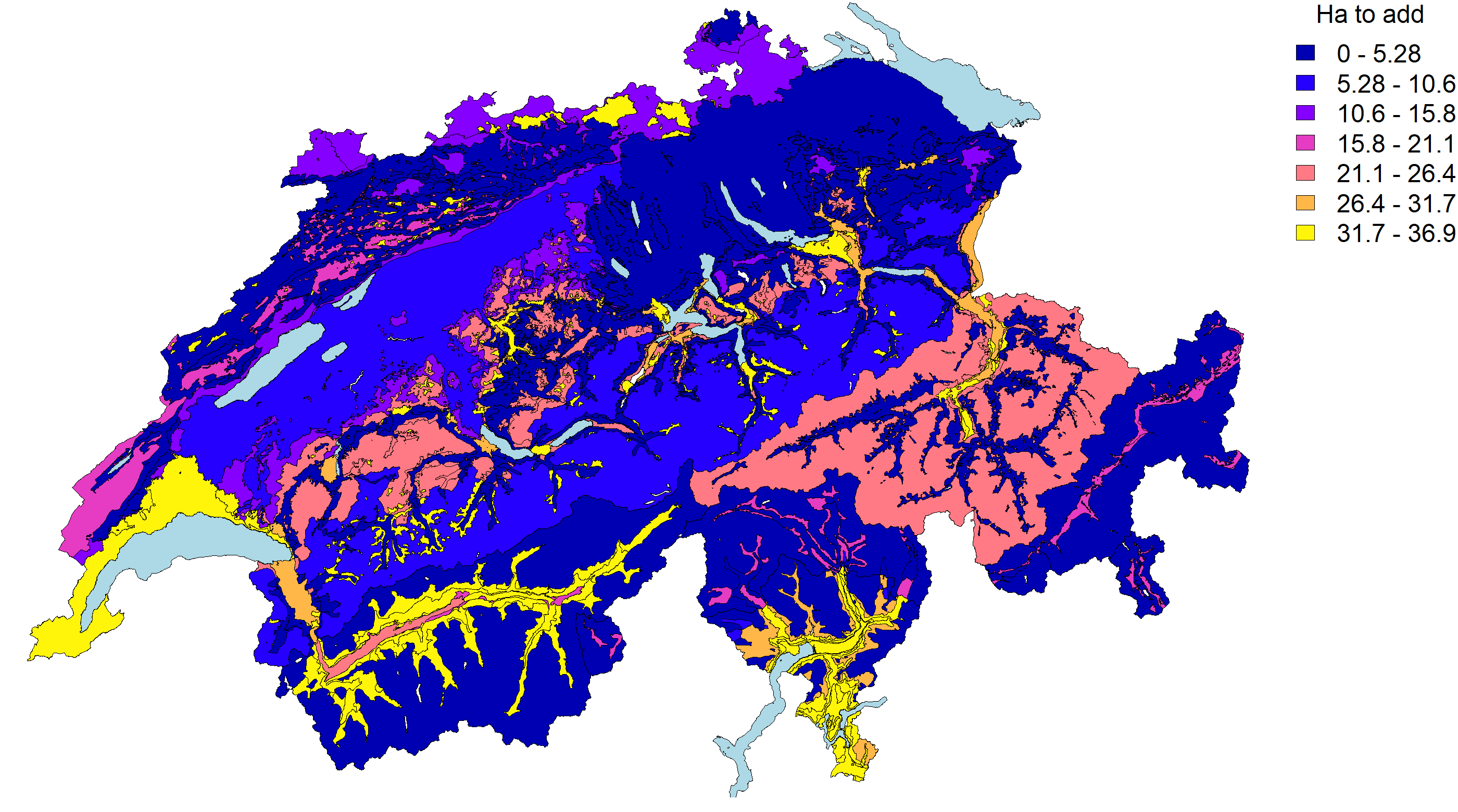

Supplement: Supplementary Figure 3 — Theoretical targeted needs at the strata level to get a distribution approaching a balanced stratified sampling over 2’750 ha. [file Image_3.png]
